# Bathyal and abyssal demersal bait-attending fauna of the Eastern Mediterranean Sea

**DOI:** 10.1007/s00227-018-3413-0

**Published:** 2018-09-21

**Authors:** Thomas D. Linley, Jessica Craig, Alan J. Jamieson, Imants G. Priede

**Affiliations:** 1 Oceanlab, University of Aberdeen, Main Street, Newburgh, Aberdeen, AB41 6AA UK; 2 Hellenic Centre for Marine Research, Box 2214, 71003 Heraklion, Crete Greece; 3 Present Address: School of Natural and Environmental Sciences, Newcastle University, Newcastle upon Tyne, NE1 7RU UK

## Abstract

Baited cameras were deployed over a depth range of 532–5111 m in the Ionian Sea to characterise the large mobile fauna. The planned installation of a neutrino telescope also offers the potential for biological observatories. The current study was intended to aid observatory placement. At increasing depths, sediment was observed to become more uniform and animal burrows and tracks reduced. A total of 10 species of deep-sea fishes were identified from images; four elasmobranchs, which were not recorded deeper than 1841 m, and six teleosts. At depths > 3000 m, including Calypso Deep, the deepest point in the Mediterranean, only one fish species was observed; the Mediterranean grenadier, *Coryphaenoides mediterraneus* (3400–5111 m), extending this species’ maximum recorded depth to 5111 m. Four species of decapod crustacea could be identified from images. The dressed deep-sea shrimp, *Acanthephyra eximia* (1346–5111 m) was the only invertebrate recorded at abyssal depths, including the deepest point. A faunal change was detected at ~ 1000 m depth. Incorporating other studies from the Eastern Mediterranean identified additional faunal boundaries at ~ 1500 m and ~ 2500 m. The time from landing the observation equipment to the arrival of the first fish increased exponentially with depth at a slower rate to that observed in the Atlantic Ocean. The estimated density of bait-attending deep-sea fish was, therefore, significantly impoverished compared to the Atlantic Ocean at equivalent depth. Barriers to colonisation, low resource input, and high temperature at depth relative to the Atlantic Ocean are probable causes of the impoverished fauna.

**Electronic supplementary material:**

The online version of this article (10.1007/s00227-018-3413-0) contains supplementary material, which is available to authorized users.

## Introduction

**Fig. 1:**
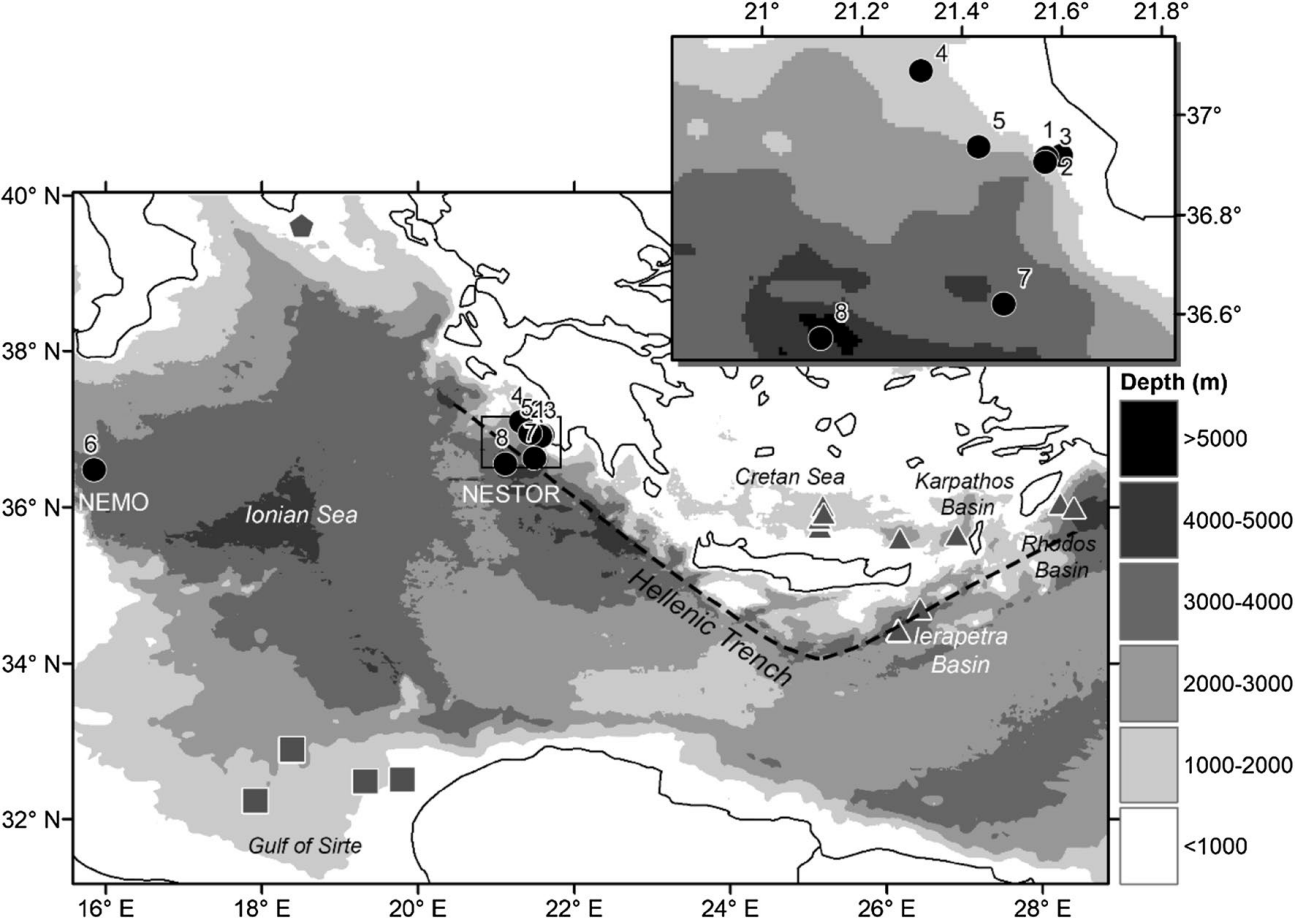
Baited lander deployments in Eastern Mediterranean Sea, black circles—present study in the Ionian Sea numbered as they appear in Table 1. Insert is a closeup of the deployments at the NESTOR site. Grey symbols denote the previous studies included in analysis; triangles—Jones et al. (2003), pentagons—Linley (2012), squares—Dan O. Jones, Andrew Gates and Jessica Craig. 2014 unpublished data. NEMO and NESTOR are locations of deep-sea neutrino telescope experiments

The Eastern Mediterranean Sea extends down to over 4000 m depth in a series of fore-arc basins sometimes known collectively as the Hellenic Trench (Fig. 1) associated with the Hellenic Arc subduction zone, where the African plate descends beneath the European tectonic plates (Royden and Papanikolaou 2011). These deep basins constitute the largest area of warm abyssal ocean (depth > 3000 m) on the planet, where the deep-sea temperature is 13–14 °C (Roether et al. 1996), compared with typical temperatures of 2–4 °C in the major oceans (Thistle 2014). This is considered similar to conditions that prevailed globally 100 million years ago during the Cretaceous, before deep-sea cooling was established (Priede 2017).

There has been little faunal continuity in the Mediterranean Sea since the Cretaceous. The majority of life in the Mediterranean was extinguished 6 million years ago when the sea dried out during the Messinian salinity crisis (Hsü et al. 1973). The present day fauna of the Mediterranean is largely the result of recolonization from the Atlantic Ocean (Pérès 1985; Bouchet and Taviani 1992) following the Zanclean flood that refilled the sea at the end of the Miocene, 5.33 million years ago (Garcia-Castellanos et al. 2009). A small number of endemic species potentially survived the salinity crisis in isolated areas with riverine input (Pérès 1985; Ryan 2009). The Mediterranean deep-sea fauna is, therefore, impoverished relative to Western Europe Atlantic slope (Pérès 1985; Bouchet and Taviani 1992; Stefanescu et al. 1992); for example, there are no deep-sea anglerfishes (Lophiiformes, Ceratioidea; Pietsch 2009) or decapods of the Glyphocrangonidae, Lithodidae, and Chirostylidae, which are characteristic deep-sea families elsewhere (Cartes 1993). Recolonization from the Atlantic is further limited by the Strait of Gibraltar, which connects the Atlantic to the Mediterranean. At only 280 m at its deepest point, potential colonisers must have at least a life history stage that allows them to pass through this shallow opening (Bouchet and Taviani 1992). It has been argued that the Mediterranean does not possess a true abyssal fauna and that the Strait of Gibraltar and high temperatures at depth may prevent colonisation from the Atlantic by abyssal species (Pérès 1985; Cartes 1993). The Mediterranean appears to have a rather high proportion of eurybaric species, unhindered by the reduction in temperature usually associated with increasing pressure (Pérès 1985; Cartes 1993).

Low-surface productivity further constrains Mediterranean deep-sea fauna, particularly in the Eastern Mediterranean, where chlorophyll concentrations are < 0.15 mg m^−3^ compared with 0.2–0.5 mg m^−3^ in the western Mediterranean (D’Ortenzio and Ribera d’Alcalà 2008) and 1.67 mg m^−3^ in the NE Atlantic (Raitsos et al. 2005). Low productivity creates a corresponding deficiency in export of organic matter from the surface to the deep sea in the Mediterranean (Rex and Etter 2010) and it has been suggested that the majority of organic input to the Mediterranean deep sea may be from terrestrial sources (Fanelli et al. 2011). Organic carbon availability is further reduced by high prevailing temperatures that accelerate microbial decomposition, potentially diminishing the fraction of surface productivity that reaches the deep sea (Laws et al. 2000), and increasing the metabolic rate of the animals that live there (Clarke 2004; Seibel and Drazen 2007). The deep-sea fish biomass in the Eastern Mediterranean is very low, D’Onghia et al. (2004) estimated 0.034 kg 1000 m^−2^ at mid slope depths (800–1300 m) in the Ionian Basin, 0.22 kg 1000 m^−2^ in the western Mediterranean, whereas Bergstad et al. (2012) reported values of 2–10 kg 1000 m^−2^ at the similar depths around the North Atlantic.

Surveys of mobile fauna from areas surrounding the current study have been performed using a variety of methods. In trawl surveys of the Ionian Sea, D’Onghia et al. (2004) recorded 44 species at depths from 600 to 4000 m and Mytilineou et al. (2005) recorded 83 demersal species living between 300 and 1200 m depth on the slopes off mainland Greece. Gates et al. (2012) conducted R.O.V surveys using both fixed baits and line transects at 2720 m depth off northern Egypt and Jones et al. (2003) made baited trap and camera observations down to 4264 m depth off the Islands of Crete and Rhodos (Fig. 1). The aforementioned studies identified the macrourid *Coryphaenoides mediterraneus* (Mediterranean grenadier) as the dominant or only fish species recorded at abyssal depths. Despite its name, *C. mediterraneus* is found throughout the North Atlantic in addition to the Mediterranean Sea (Geistdoerfer 1986). It is an active predator and feeds mainly on peracarid crustaceans on or just above the sediment (Carrassón and Matallanas 2002; Gates et al. 2012; Pérez-i-García et al. 2017), and while it gathers at baited experiments, it has not been observed directly feeding on the bait (Jones et al. 2003; Gates et al. 2012). The only other fish species observed by Gates et al. (2012) and collected at abyssal depths by D’Onghia et al. (2004) were the ipnopid (deep-sea tripod fish) *Bathypterois dubius*, a sit-and-wait predator which is unlikely to be attracted to bait, and the rare Bythitid (viviparous brotula) *Cataetyx laticeps* which also does not appear to respond to bait (Gates et al. 2012; Jamieson et al. 2017). *Lepidion lepidion* (Mediterranean codling) was identified as one of the dominant fish between 1000 and 1400 m depth in the Mediterranean Sea (Stefanescu et al. 1993; D’Onghia et al. 2004). Only four shark species are known to live exclusively below 1000 m in the Mediterranean Sea (Sion et al. 2004) and sharks have only been reported in the Mediterranean to ~ 2800 m depth (Jones et al. 2003; Sion et al. 2004; Gates et al. 2012). The deepest reported shark species in the Mediterranean is *Centroscymnus coelolepis* (Portuguese dogfish; 1500–2800 m); however, it appears to have only colonised the western Mediterranean (Carrassón et al. 1992; Sion et al. 2004). Sharks may be physiologically or energetically restrained from occurring deeper (Priede et al. 2006; Treberg and Speers-Roesch 2016).

The decapod fauna found by Cartes (1993) in the western Mediterranean was similar to that found in the Atlantic. However, a small number of tropical species, *Aristeus antennatus*, *Acanthephyra eximia*, and *Plesionika acanthonotus*, are far more dominant in the Mediterranean community than they are in the North Atlantic. Cartes (1993) found them to be common down to depths of about 2000 m, with *A. exima* extending further, to the deepest areas sampled (2261 m). *Aristeus antennatus* (blue and red shrimp) is a large and abundant shrimp in the Mediterranean and as a result is not only of commercial interest but also of ecological importance (Pérès 1985; Cartes 1994). It is a predator with a varied diet which may also exploit carrion as a passive scavenger (Cartes 1994).

The present study was undertaken as part of an evaluation of potential sites for the installation of biological observatories incorporated into astronomical neutrino observatories (Feder 2002) as part of the KM3NeT project (Carr et al. 2008) in the deep waters of the Ionian Sea. The Mediterranean Sea is an excellent location of such a neutrino telescope, the water has high transparency, there are areas of very deep water close to land, complete sky coverage is possible in conjunction with other global neutrino detectors, and the Mediterranean Sea faces the galactic centre two-thirds of the time (Circella 2009). Three first generation telescopes were installed in the Mediterranean sea; Antares off the Southern French coast (Spurio 2002; Circella 2009), NEMO close to the Sicilian coast (Taiuti et al. 2011) and NESTOR off the south-eastern tip of the Peloponnese near the Calypso Deep, in the vicinity of the town of Pylos (Rapidis 2009: Fig. 1). The installation of a neutrino telescope requires seabed modular junction boxes, which provide instrument power and data transfer to shore. Once this infrastructure is in place, additional sensors connected to these junction boxes are relatively inexpensive and expand the project into a multidisciplinary endeavour.

To identify locations of biological interest, where a permanent biological observatory could be placed, a series of baited camera lander deployments were made during 2008–2011 at the NESTOR site at depths from 532 m down to 5111 m and one deployment near the NEMO site in the West at 3396 m. The additional aims of this study were to determine whether there is active life at the maximum depth of the Mediterranean Sea, and to identify the species present, while also comparing the abundance of deep-sea fishes in the Mediterranean Sea with the Atlantic Ocean.

## Materials and methods

### Baited landers

Baited photographic landers were first developed in 1969, specifically to study deep-sea mobile faunal (Isaacs and Schwartzlose 1975). The method is particularly suited to deep-sea research as the cost and time benefits relative to other survey methods become more pronounced with increasing depth (Jamieson 2016). Luring animals with bait to a camera helps to amplify the low density of deep-sea mobile faunal and is particularly adept at recording large mobile carnivores that are often able to avoid other survey methods (Bailey et al. 2007; Harvey et al. 2007). The method emulates a natural process, the arrival of a carcass at the seabed, which is distinct from the input of particulate material from the surface (Mahaut et al. 1990; Drazen et al. 2008; Higgs et al. 2014). The method is highly selective, however, and the proportion of the mobile fauna that respond to bait varies with location and depth (Priede and Merrett 1996; Yau et al. 2001) and is likely related to how regularly that location experiences a natural food fall (Cartes et al. 2016). The fauna which respond to baited systems are often referred to as scavengers; however, bait-attending fauna is a more accurate collective term for two trophic guilds recognised by Gartner et al. (1997); the necrophages or scavengers that directly consume dead material and necrophagivores that feed on the necrophages, as well as other species that may use the carcase as habitat without consuming any material. Species such as the European conger (*Conger conger*) intercept the bait rapidly and are seen to feed directly upon it, classifying them as necrophages when bait-attending (Bozzano and Sardaà 2002; Castro et al. 2005; Linley et al. 2017b). This does not necessarily mean that carrion forms an important component of *C. conger* diet, rather that it is attracted to bait to feed upon it. Others rely on larger scavengers to perturb the bait before feeding, such as arrowtooth eels (Jamieson et al. 2011). Necrophagivores are attracted to bait to exploit the higher density of prey species, often amphipods, in the vicinity (Bozzano and Sardaà 2002; Castro et al. 2005; Kemp et al. 2006; Stoner et al. 2008; Linley et al. 2016, 2017a; Drazen and Sutton 2017), although they may use bait odour to locate the prey. Other species such as the blackbelly rosefish and some Zoarcid species may use the associated structures as a habitat as well as hunting grounds (Higgs et al. 2014; Jamieson et al. 2017; Linley et al. 2017b). For these reasons, the fauna observed by baited camera deployed using the lander methods are henceforth described as ‘bait-attending’.

Data were collected using two baited landers based on the ROBIO design (Jamieson and Bagley 2005) over three cruises using the *RV Philia* (HCMR, Greece), *RV Pelagia* (NIOZ, The Netherlands) and *FS Meteor* (Germany; Table 1).

**Table 1:** Baited lander deployments within the Ionian Sea numbered in order of increasing depth

Deployment no.	Depth (m)	Location	Vessel	Date	Duration (h)
1	532	36.91761°N, 21.59818°E	Philia	15/10/2008	6
2	737	36.91420°N, 21.56850°E	Philia	14/10/2008	1.8
3	943	36.90412°N, 21.56541°E	Philia	16/10/2008	3.6
4	1346	37.08767°N, 21.31767°E	Meteor	02/02/2011	4
5	1841	36.93517°N, 21.43233°E	Meteor	28/01/2011	3.2
6	3396	36.47450°N, 15.84533°E	Meteor	24/01/2011	1
7	4204	36.61898°N, 21.48337°E	Pelagia	14/12/2009	17.3
8	5111	36.55050°N, 21.11617°E	Meteor	30/01/2011	4.1

Location is given in decimal degrees. The duration is the amount of time recorded on the seabed

All lander deployments at less than 1000 m depth used the baited camera orientated horizontally, focussed at bait mounted on a rigid arm 90 cm in front of the camera. Animals were, therefore, photographed in profile. Bait was a locally sourced whole mackerel (*Scomber* spp.). The scientific payload included a temperature and pressure sensor (UCM-60; Sensortec, Norway) with a 30 s sample interval. All deployments > 1000 m was conducted with the lander connected to the ballast weight, with bait attached, via a 2 m metal strop to suspend the lander 2 m above the seabed with the camera facing downwards, so that animals were viewed from above. The tethered lander also measured conductivity, temperature, pressure, current speed, and direction throughout at 5 min intervals using a Seaguard system (Aanderaa Instruments, Norway).

The landers were deployed by free-fall from the ship and, following arrival on the seabed, a digital stills camera (OE14-208, Kongsberg Maritime, Norway) with white light strobe (OE11-242, Kongsberg Maritime, Norway) was programmed to take an image every minute. Images were JPEG; 2592 × 1944 pixels with a field of view of approximately 3.4 m^2^ in the deeper vertical images and 2.6 m wide on the > 1000 m depth horizontal images.

### Species identity and indicators of local density

The camera resolution was sufficient to identify all species larger than approximately 2 cm total length, omitting amphipods, isopods, and mysids from the analysis. Species identification was aided by reference to the literature (Campagno 1984; Whitehead et al. 1986; Cohen et al. 1990; Wilson et al. 1996; Ebert and Stehmann 2013; Daly-Engel et al. 2018) and previous lander images of known species (Jones et al. 2003; Bailey et al. 2005; Linley et al. 2017b). *Coryphaenoides mediterraneus* was distinguished from *C. guentheri* by its more rounded snout and iridescent appearance (Gates et al. 2012) supported by voucher specimens captured by Jones et al. (2003) and Bailey et al. (2003). As mentioned by Gates et al. (2012), there is the possibility that *C. guentheri* is also present in the minority; however, it was not positively identified in any of the images.

For each species, the first arrival time (*t*_arr_) was determined by the number of minutes elapsed from bait arrival on the sea floor until the first individual appears within the field of view of the camera. The maximum number of a given species (*N*_max_) was the most observed simultaneously and % images are the percentage of images in which a given species was observed. Data were combined with comparable studies from around the study site; from Santa Maria de Luca off the coast of southern Italy (Linley et al. 2017b), the Cretan Sea (Jones et al. 2003), and the Gulf of Sirte (Dan O. Jones, Andrew Gates and Jessica Craig. 2014 unpublished data; Fig. 1).

### Statistical analysis

Multivariate analysis was conducted in PRIMER v 7.0.11 (Clarke and Gorley 2015) on square root transformed *N*_max_ data. This transformation was selected to reduce the influence of dominant species, but was not excessively powerful as species counts tended to be low. A resemblance matrix was formed based on Bray–Curtis similarity. SIMPROF (similarity profile permutation test) analysis was used to identify the significant groups within the ecological data and these groups were further validated with one-way ANOSIM (analysis of similarities) and visualised via CLUSTER (dendrogram of hierarchical grouping of samples) analysis. SIMPER (Similarity percentage) analysis identified the species which drove the intra group similarity. The current study was compared with the previous studies which had used the same methodology. Abiotic data; depth, latitude, longitude, and the duration of the deployment, were compared to identify any differences between the studies/basins. However, there was not sufficient replication to allow distinction between the multiple environmental gradients which exist between the studies, e.g., season, year, temperature, productivity, fishing pressure, etc. LINKTREE (linkage tree) analysis identified the abiotic variables which correlated with the significant faunal divisions identified in the CLUSTER analysis.

### Density estimation

To compare the bait-attending Eastern Mediterranean Sea deep-sea demersal fish density to the Atlantic Ocean, *t*_arr_ of the first fish to arrive of any species was combined from studies using comparable lander configuration. The Mediterranean data set was compared against studies from the Atlantic Ocean (Armstrong et al. 1992; Priede et al. 1994; Smith et al. 1997; Henriques et al. 2002; King et al. 2006; Cousins et al. 2013; Jamieson et al. 2017). In both locations, *t*_arr_ increased logarithmically (base 10) with increasing depth in accordance with the fitted relationship shown in the following equation:

1 $${\text{Log}}_{10} \left( {t_{\text{arr}} } \right) = a + {\text{Depth}} \;({\text{m}})\; \times \;b,$$

where *a* and *b* are constants. Comparisons between the Mediterranean Sea and the Atlantic Ocean were tested via ANCOVA of *t*_arr_, with “depth” as a covariate and “location” as a factorial explanatory variable. Statistical analysis was conducted in R (R Development Core Team 2005) and plots were made using ggplot2 (Wickham and Chang 2007).

To illustrate how *t*_arr_ relates to fish population density, the theoretical fish density (*A*, km^−2^) was calculated using the equations from Priede et al. (1990):

2 $$r = \frac{{t_{\text{arr}} }}{{\left( {\frac{1}{{V_{\text{f}} }} + \frac{1}{{V_{\text{w}} }}} \right)}}$$

3 $$A = \frac{{10^{6} }}{{3 r^{2} }}$$

where *r* is the radius of the space occupied by each fish, *V*_f_ is the average speed of the fish over the ground, and *V*_w_ is the speed of dispersal of the odour plume on the bottom current. It is assumed that *V*_f_ = *V*_w_ = 0.05 m s^−1^, typical of deep ocean conditions (Priede et al. 1990). *t*_arr_ is first arrival time expressed in seconds.

## Results

**Fig. 2:**
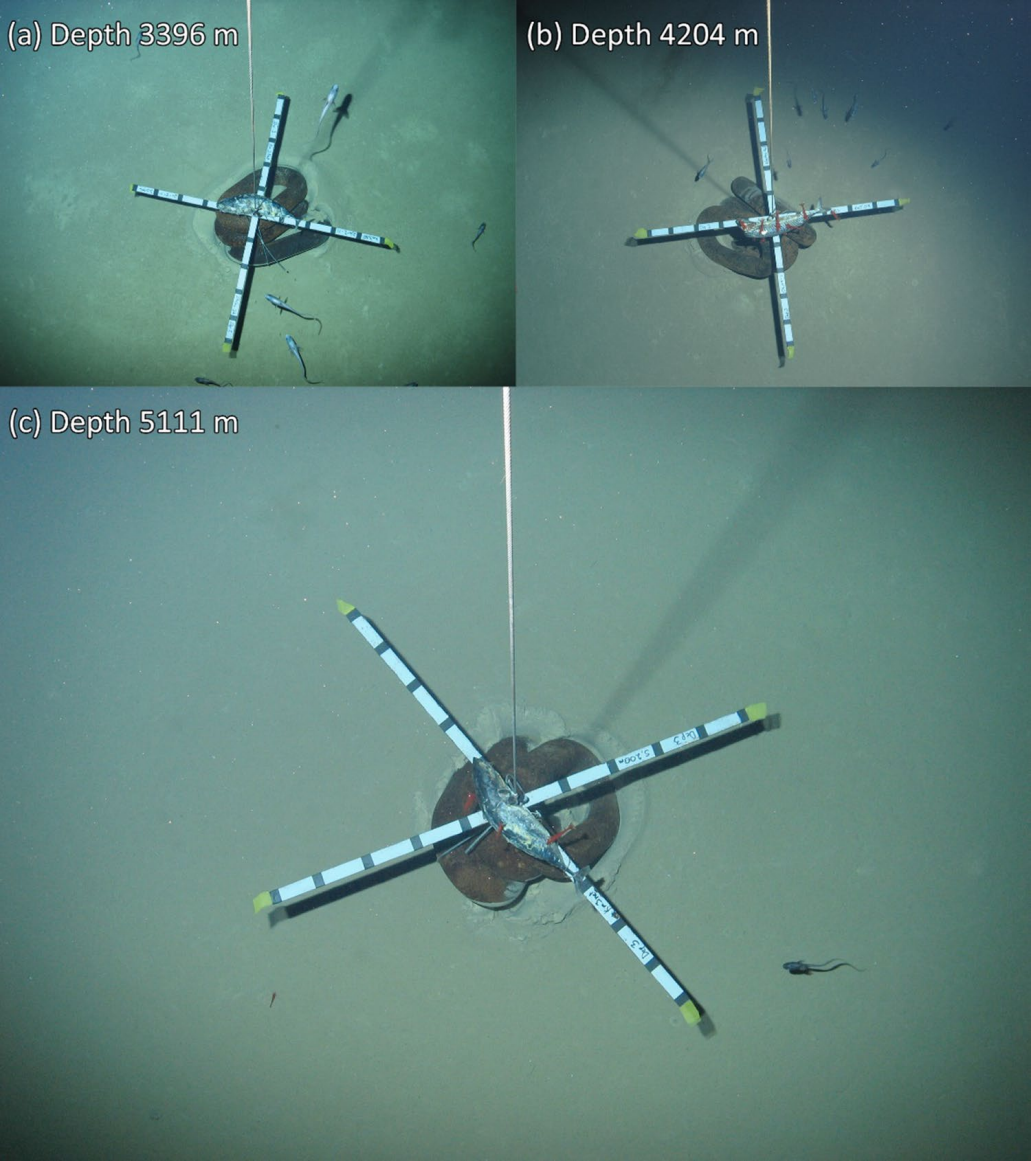
Downward-looking views from the tethered lander of the ballast, bait and scale cross (image centre), approaching fauna and the surrounding sediment at abyssal depths in the Ionian Sea. **a** is in the western Ionian Sea, **b** and **c** are in the eastern Ionian Sea. Evidence of animal tracks and bioturbation diminish with increasing depth until appearing completely smooth and uniform at the deepest deployment (**c**)

**Fig. 3:**
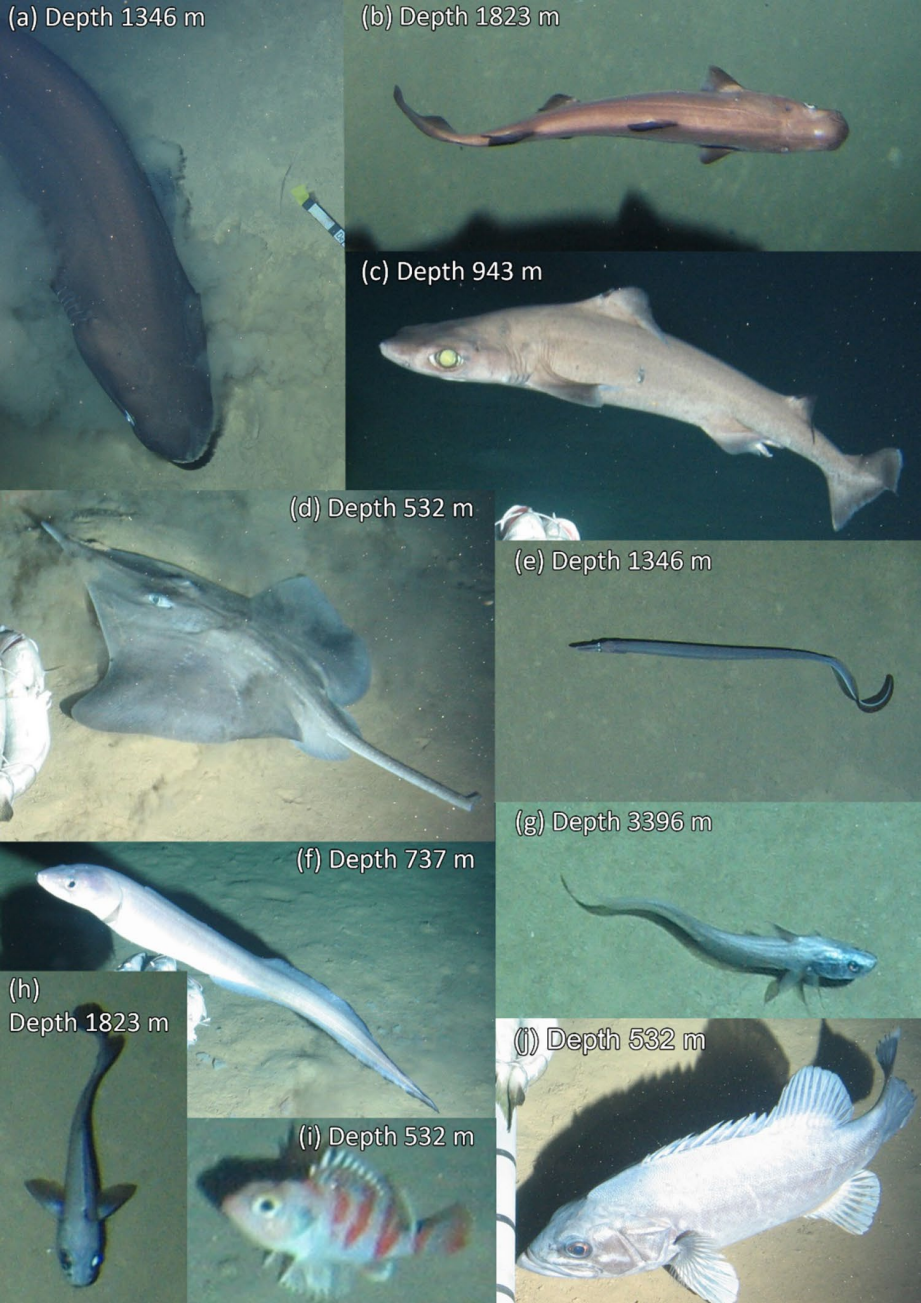
Observed fish species. Identity is given as scientific name (Family, common name); the sharks, **a** *Hexanchus griseus* (Hexanchidae, bluntnose sixgill shark), **b** *Etmopterus spinax* (Etmopteridae, velvet belly shark), and **c** *Centrophorus granulosus* (Centrophoridae, gulper shark), **d** the ray *Dipturus oxyrinchus* (Rajidae, longnosed skate), the eels, **e** *Nettastoma melanurum* (Nettastomatidae, blackfin sorcerer) and **f** *Conger conger* (Congridae, European conger), the Gadiformes, **g** *Coryphaenoides mediterraneus* (Macrouridae, Mediterranean grenadier) and **h** *Lepidion lepidion* (Moridae, Mediterranean codling), the Scorpaeniformes, **i** *Helicolenus dactylopterus* (Sebastidae, blackbelly rosefish) and the Perciformes, **j** *Polyprion americanus* (Polyprionidae, wreckfish)

**Fig. 4:**
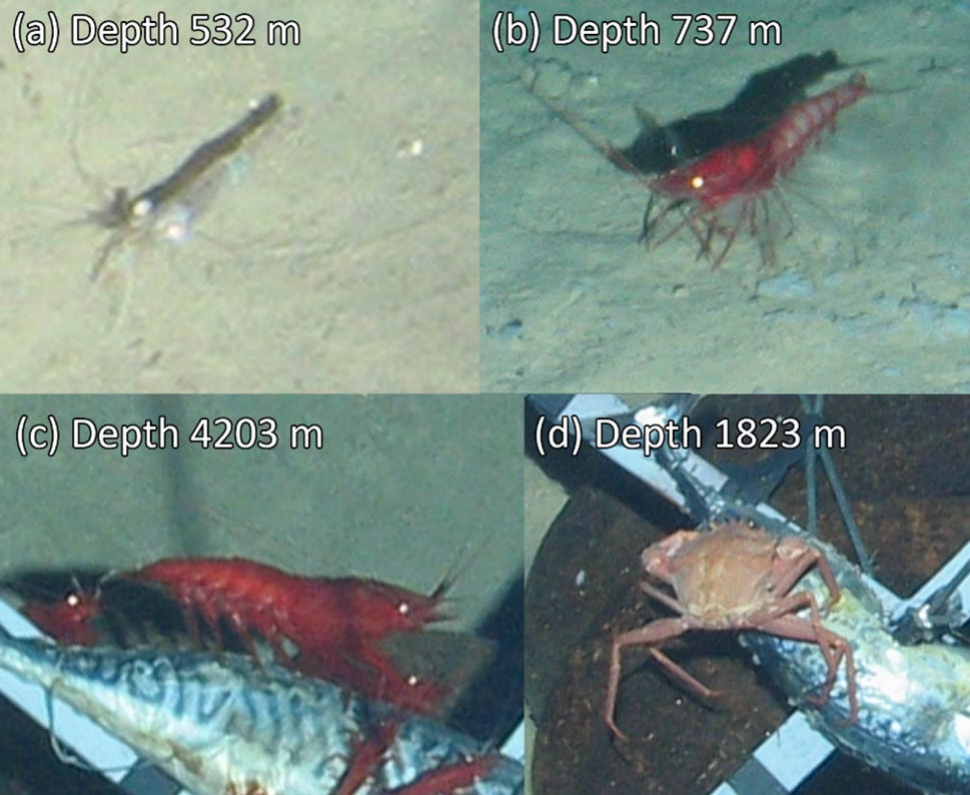
Observed invertebrate species were all decapods. Identity is given as scientific name (Family, common name); **a** *Plesionika heterocarpus* (Pandalidae, arrow shrimp), **b** *Aristeus antennatus* (Aristeidae, blue and red shrimp), **c** *Acanthephyra eximia* (Acanthephyridae, dressed deep-sea shrimp), **d** *Chaceon mediterraneus* (Geryonidae)

The baited lander system was successfully deployed eight times from 532 to 5111 m water depth. Almost two and a half thousand seabed images were taken. The seabed at all deployments was open sandy sediment without visible hard surfaces or complex habitats. In the Calypso Deep, the seabed was exceptionally uniform with no visible biologically formed changes to the sediment, e.g. tracks, burrows, faecal casts, etc. (lebensspuren). The three deepest deployments showed increasingly uniform and clean sediment (Fig. 2). Table 2 gives the recorded environmental conditions and bait-attending fauna identified. Temperature varied between 13.8 and 14.3 °C and appeared to get warmer with increasing depth beyond ~ 2000 m. Salinity was stable at 38.7 PSU. Current speeds were generally low, < 10 cm s^−1^, but an average current speed of 17.9 cm s^−1^ was recorded at 4202 m depth (no. 7), more than double any other measurement. Ten fish species (Fig. 3) and four invertebrate species (Fig. 4) could be identified from the lander images.

**Table 2:** Environmental conditions and bait-attending animals recorded during deployments numbered in increasing depth order as in Table 1 and Fig. 1

Deployment no.	1	2	3	4	5	6	7	8
Depth (m)	532	737	943	1346	1823	3396	4204	5111
Current (cm s^−1^)				2.7 ± 0.7	5.7 ± 1.5	7.9 ± 1.2	17.9 ± 1.8	2.4 ± 0.82
Temperature (°C)	14.2	13.9	13.8	13.8	13.8	14	14.2	14.3
Salinity (PSU)				38.7	38.7	38.7	38.7	38.7
Elasmobranchii								
*Hexanchus griseus* (bluntnose sixgill shark)				**1** (116) 6.2				
*Etmopterus spinax* (velvet belly shark)					**2** (4) 9.4			
*Centrophorus granulosus* (gulper shark)	**1** (99) 3.1	**2** (28) 31.5	**3** (14) 25.0					
*Dipturus oxyrinchus* (longnosed skate)	**1** (49) 1.9							
Teleostei								
*Nettastoma melanurum* (blackfin sorcerer)				**3** (58) 19.8				
*Conger conger*—(European conger)		**1** (4) 6.5						
*Coryphaenoides mediterraneus* (Mediterranean grenadier)						**7** (8) 79.6	**8** (67) 84.9	**1** (174) 0.4
*Lepidion lepidion* (Mediterranean codling)				**1** (55) 2.1	**2** (76) 8.4			
*Helicolenus dactylopterus* (blackbelly rosefish)	**1** (23) 27.5	**1** (91) 4.6						
*Polyprion americanus* (wreckfish)	**2** (63) 34.4							
Crustacea: Decapoda								
*Plesionika heterocarpus* (arrow shrimp)	**4** (1) 28.7							
*Aristeus antennatus* (blue and red shrimp)	**3** (31) 7.2	**2** (19) 10.2						
*Acanthephyra eximia* (dressed deep-sea shrimp)				**7** (12) 59.5	**14** (2) 89.0	**2** (6) 13.0	**29** (3) 99.4	8 (**1**) 22.1
*Chaceon mediterraneus*					**1** (156) 12.6			

Each identified species is reported as: maximum number in a single image (in bold), (time of first arrival in minutes), % of seabed images observed in

### Community structure

**Fig. 5:**
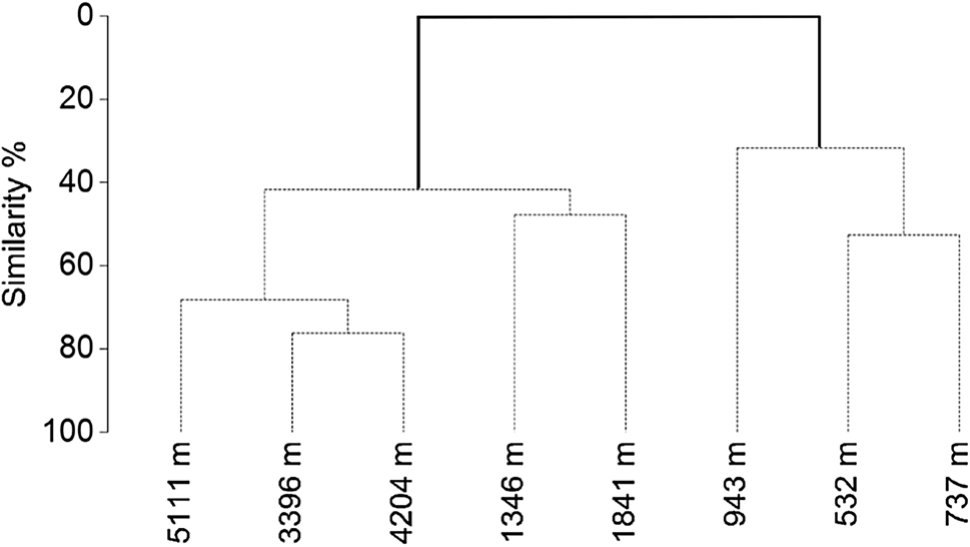
Dendrogram expressing faunal similarity between deployments. Deployments are labelled by their depth (m). Solid line indicates significant (*p* = 0.018) faunal division identified by SIMPROF and confirmed through ANOSIM

There was no species overlap between the three deployments at < 1000 m and those > 1000 m depth. As a result, SIMPROF analysis (Fig. 5) identified a significant faunal divide between deployments shallower and deeper than ~ 1000 m (confirmed by ANOSIM; *R* = 1, *p* = 0.018). SIMPER analysis identified those species which contributed the most to the within group similarity (Table 3). All deployments within the shallower groups contained the gulper shark *Centrophorus granulosus*, while all deeper deployments contained the shrimp *Acanthephyra eximia*. Deployments between 1000 and 2000 m depth would also have no species overlap with those > 2000 m, if not for the inclusion of the shrimp *Acanthephyra eximia* in all deployments > 1000 m. No elasmobranch species were reported beyond 1841 m depth. Only two species were observed in the three deepest deployments, *A. exima* and the macrourid *Coryphaenoides mediterraneus*. At 5111 m deep in the Calypso Deep, the deepest point in the Mediterranean Sea, *C. mediterraneus* arrived within the field of view after 2.9 h and only one individual was seen for a brief period of time. *Acanthephyra eximia* was immediately present on landing but present in fewer images (22.1%) and reached a lower maximum number (8) than at 4203 m, where *N*_max_ (29) and % of images (99.4%) would indicate it was most abundant.

**Table 3:** Similarity percentage analysis (SIMPER) of the species which contributed to the similarity within the identified faunal groups

	Average *N*_max_	Average similarity	Percentage contribution
Shallower sites—532–943 m. Average similarity: 38.66%			
*Centrophorus granulosus*	1.38	26.26	67.92
*Aristeus antennatus*	1.05	7.27	18.79
*Helicolenus dactylopterus*	0.67	5.14	13.29
Deeper sites—1346–5111 m. Average similarity: 51.06%			
*Acanthephyra eximia*	3.29	41.41	81.11
*Coryphaenoides mediterraneus*	1.33	8.17	16
*Lepidion lepidion*	0.48	1.48	2.89

Average *N*_max_, average similarity and each species’ percentage contribution to the within group similarity are presented

### Comparable Mediterranean studies

**Fig. 6:**
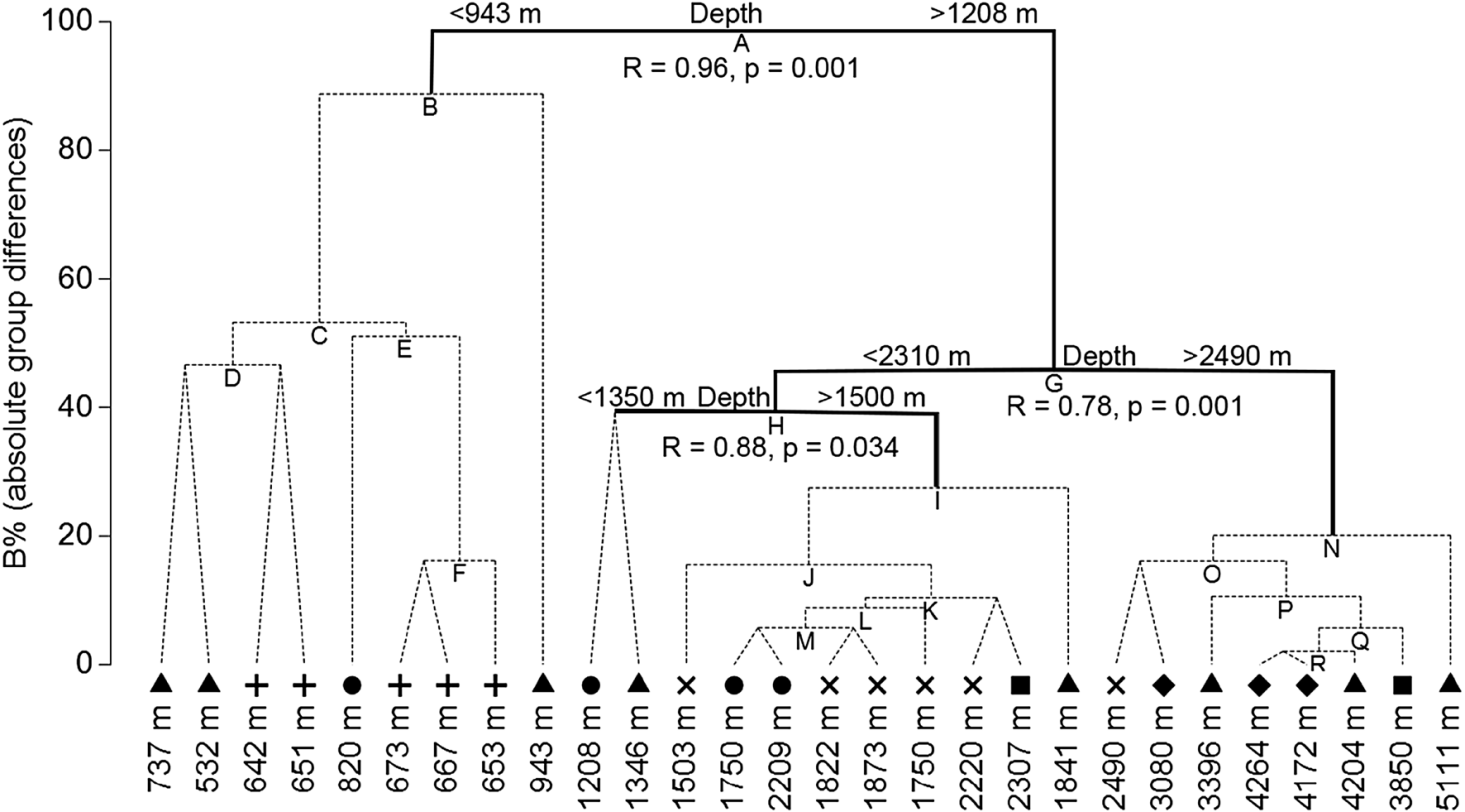
LINKTREE analysis of Eastern Mediterranean baited lander Studies. Deployments are labelled by depth with symbols denoting location: Triangle—the current study in the Ionian Sea, cross—Cretan Sea, square—Rhodos Basin and diamond—Ierapetra Basin from Jones et al. (2003), circle—Gulf of Sirte from Dan O. Jones, Andrew Gates and Jessica Craig (2014) unpublished data and plus—Santa Maria de Luca from Linley (2012). Thick lines indicate significant (*p* = 0.001) divides in the fauna identified by SIMPROF and confirmed through ANOSIM, which are annotated with their correlations with the abiotic data

Studies using very similar sampling methodology have been conducted at sites around the current study (Fig. 1). An Eastern Mediterranean data set was created from Jones et al. (2003) to the East of the current study, Linley (2012) from the northwest and Dan O. Jones, Andrew Gates and Jessica Craig (2014) unpublished data from the southwest. Including these studies identified four significant faunal groups which appeared to be organised by depth (groups were identified through SIMPROF analysis and verified through ANOSIM; *R* = 0.769, *p* = 0.001). LINKTREE analysis indicated that all the significant faunal divides identified were driven solely by depth (or by a factor correlated with depth; Fig. 6). SIMPER analysis was used to identify the species which contributed most to the similarity within the identified groups (Table 4). A complete list of the SIMPER and LINKTREE analysis is included in the supplementary material.

**Table 4:** Similarity percentage analysis (SIMPER) of the species which contributed to the similarity within the identified faunal groups of the combined Eastern Mediterranean data set up to 70% of the cumulative within group similarity

	Average *N*_max_	Average similarity	Percentage contribution
Depth < 943 m. Average similarity: 32.32%			
*Aristeus antennatus*	0.95	7.81	24.16
*Conger conger*	0.82	7.60	23.52
*Plesionika heterocarpus*	0.84	4.59	14.20
*Helicolenus dactylopterus*	0.60	3.69	11.42
Depth 1208–1346 m. Average similarity: 66.50%			
*Acanthephyra eximia*	2.53	29.93	45.01
*Nettastoma melanurum*	1.73	23.18	34.86
Depth 1503–2307 m. Average similarity: 66.56%			
*Acanthephyra eximia*	8.67	39.80	59.80
*Etmopterus spinax*	2.24	12.19	18.32
Depth > 3396 m. Average similarity: 67.24%			
*Acanthephyra eximia*	4.32	39.63	58.93
*Coryphaenoides mediterraneus*	2.98	25.17	37.43

Full analysis output is included in Supplementary information 1. Headings as in Table 3

### Eastern Mediterranean fish density relative to the Atlantic Ocean

**Fig. 7:**
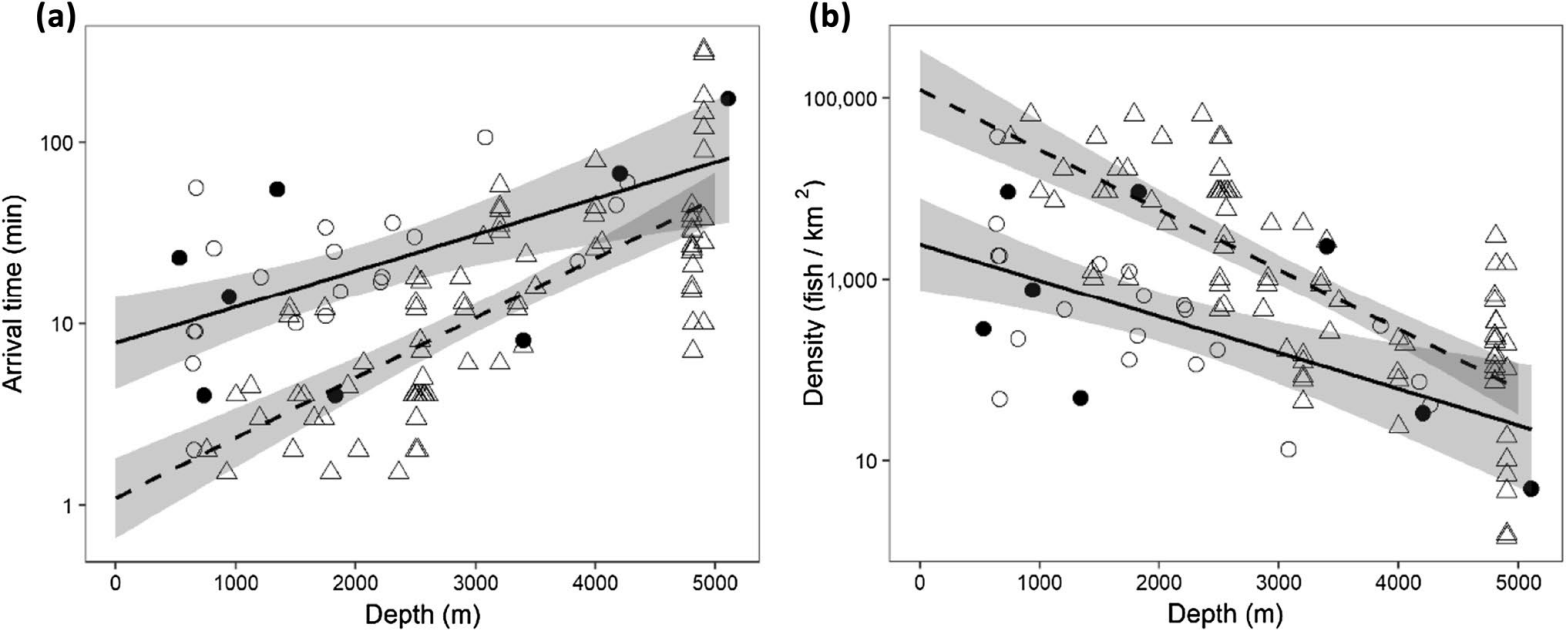
Comparison of depth-related trends in fish arrival times and theoretical density in the Eastern Mediterranean and the Atlantic. **a** First fish arrival times (*t*_arr_) against depth. **b** Shows the estimated density of fishes calculated from the first arrival times (Priede et al. 1990). Data from the Eastern Mediterranean (circle, filled circles from this study, with solid regression line), data from the Atlantic (triangle, dashed regression line). Shaded area represents the 95% confidence interval of the model fit

The time from the arrival of the lander to the arrival of the first fish (*t*_arr_) had a significant positive relationship with depth (*F*_3,107_ = 41.18, *p* < 0.001). The Mediterranean had significantly higher intercept than the Atlantic (*F*_1,107_ = 39.716, *p* < 0.001). Interaction between location and depth was detected (*F*_1,107_ = 4.421, *p* = 0.038) indicating that the rate at which *t*_arr_ increase with increasing depth was greater in the Atlantic (Fig. 7a). At 1000 m depth, the estimated time taken for the first fish to arrive at a baited lander in the Atlantic ocean was one-fifth that of the Eastern Mediterranean (2.4 vs. 12.3 min), but at 4000 m, the Atlantic was approximately half the Mediterranean (23.0 m vs. 49.1 min). The two regression lines (equations given in Table 5) would cross at 6507 m depth, beyond the maximum depth of the Mediterranean Sea, and therefore, estimated arrival times were always longer in the Eastern Mediterranean relative to the Atlantic Ocean at equivalent depth.

**Table 5:** Equations and statistics of the regression lines plotted in Fig. 7

Arrival time (*t*_arr_)	Equation	*N*	*R* ^2^	Test statistic	*P* value
Atlantic	Log_10_(*t*_arr_ (min)) = 0.0386 + 0.000331 × depth (m)	83	0.613	*F*_3,107_ = 41.18	< 0.001
Mediterranean	Log_10_(*t*_arr_ (min)) = 0.892 + 0.000200 × depth (m)	28			
Fish density					
Atlantic	Log_10_(fish km^−2^) = 5.093—0.000662 × depth (m)	83	0.613	*F*_3,107_ = 41.18	< 0.001
Mediterranean	Log_10_(fish km^−2^) = 3.387—0.000400 × depth (m)	28			

*N* = the number of datapoints

The theoretical density of bait-attending deep-sea fishes in the Eastern Mediterranean was much lower than in the Atlantic Ocean: estimated as 972 and 27022 fish km^−2^, respectively, at 1000 m depth and 25 and 61 fish km^−2^ at 5000 m depth (Fig. 7b, Table 5).

## Discussion

This is the first study to extend baited lander observations to the deepest area in the Mediterranean Sea, allowing the study of faunal zonation from 532 to 5111 m depth in the Eastern Mediterranean. All the fish species found in the present study have been recorded in the previous Ionian Sea trawl surveys (D’Onghia et al. 2004; Mytilineou et al. 2005) with the exception of the Longnose skate *Dipturus oxyrinchus,* which is recorded from the Western Mediterranean (Griffiths et al. 2011), the Aegean Sea (Yigin and Ismen 2010) and the Gulf of Sirte (Dan O. Jones, Andrew Gates and Jessica Craig. 2014 unpublished data). D’Onghia et al. (2012) found that that *Conger conger*, *Helicolenus dactylopterus* and *Polyprion americanus* generally occur in higher abundance in cold-water coral areas than on open slopes of the Northern Ionian Sea at 450–650 m depth. The presence of the shark *Etmopterus spinax* is consistent with the depth range 1500–2300 m in the Cretan and Levantine Seas (Jones et al. 2003), 820–2209 m in the Gulf of Sirte, Ionian Sea (Dan O. Jones, Andrew Gates and Jessica Craig. 2014 unpublished data) and 600–2200 m reported from the Eastern Ionian Sea, near the site of the current study (Sion et al. 2004).

A distinct boundary in the bait-attending assemblage was found at ~ 1000 m depth. The shark *Centrophorus granulosus*, the decapod *Aristeus antennatus* and the scorpaeniform *Helicolenus dactylopterus* were exclusively found at the shallower sites, while *Acanthephyra eximia*, *Coryphaenoides mediterraneus* and *Lepidion lepidion* were only observed at the deeper sites. When combining studies from around the Eastern Mediterranean additional boundaries were found at ~ 1500 m and ~ 2500 m, which all contained *A. exima,* but were separated by the fish *Nettastoma melanurum* and *Etmopterus spinax,* respectively. Beyond ~ 2500 m depth, only two species, *A eximia* and *C. mediterraneus*, were observed to maximum depth in the Calypso Deep.

The previous studies using different methodology have found faunal changes at similar depths within the Mediterranean Sea. Within the decapod fauna, Cartes (1993) found faunal changes between 1000 and 1200 m and at around 2000 m depth. At 1200 m depth Stefanescu et al. (1992, 1993) found a turnover in fish species, a more diverse and heterogeneous group of larger fish species with higher energetic requirements were replaced with a small number of species and homogeneous community of smaller, less active fish with lower energetic demands. The replacement of large fish species with smaller ones was not compensated by increased abundance, and as a result, this turnover also leads to a drop in overall fish biomass (Stefanescu et al. 1992). The faunal boundary at 1200–1400 m depth is thought to be the result of an abrupt reduction in available prey, namely, of mesopelagic organisms as prey items (Stefanescu et al. 1993). Deposit feeding becomes more important in decapods with increasing depth (Cartes 1998). With respect to the decapod fauna, Cartes (1993) hypothesised that the Mediterranean decapod fauna was unlikely to change significantly below 2265 m depth (the deepest sampling depth in the study) and was likely to include *Acanthephyra eximia* as a dominant species. Our study appears to support this. Cartes (1993) suggested that due to the environmental stability in the Mediterranean, the depth zonation observed in decapods was not the result of pressure tolerance but rather other factors, likely relating to food supply. For fish, there was no species turnover of bait-attending species at abyssal depths in agreement with Jones et al. (2003), and while D’Onghia et al. (2004) captured a wider variety of species via trawl, there was a single fish faunal group > 1500 m depth common to the three locations studied. It would, therefore, appear that within the large mobile fauna of the Mediterranean Sea, there is little turnover at abyssal depths and no indication of specifically abyssal large mobile fauna.

The Red Sea provides an interesting comparison to the Mediterranean Sea in which to explore how pressure, temperature and productivity effect faunal composition. Compared to the Mediterranean, the Red Sea has a more extreme environment, namely, higher temperature and increased salinity throughout the water column (Khalaf and Zajonz 2007). The Red Sea also experienced a hypersaline event, but faunal continuity was maintained by appropriate conditions in the Gulf of Aqaba and southern Red Sea (Dibattista et al. 2016). As a result, the Red Sea appears to possess a high proportion of endemism (Dibattista et al. 2016), 17% endemic deep-sea fish species (Zajonz 2007), and 30% in the invertebrates (Türkay 1996). Primary production in the Red Sea is higher than in the Mediterranean, however, at 1.69 g C m^−2^ day^−1^ rather than 0.59, respectively, for whole sea satellite-derived estimates (Longhurst et al. 1995). Similarities in conditions between the two seas have allowed the introduction of invasive species into the Mediterranean following the opening of the Suez Canal in 1869 (Ben-Tuvia 1966). Both pressure and temperature affect proteins in similar ways (Somero 2003) and a combined piezo-thermal effect may limit faunal depth ranges (Carney 2005). Carney (2005) suggested that the presence of vertical zonation of deep-sea fauna in areas of high temperature at depth may indicate that pressure is a greater adaptive barrier than temperature. Still, it would appear that adaptation to increasing pressure, without the associated decrease in temperature found in most waterbodies, is less of a barrier to shallow species being found deeper. Similar to the Mediterranean Sea, the uniform temperature throughout the water column in the Red Sea allows for wide vertical distributions in the fauna (Türkay 1996; Zajonz 2007). The Red Sea lacks what would be considered a truly deep-sea fauna, but instead, there has been a downward extension of near-surface species which enter through the shallow Strait of Bab al Mandab at its southern entrance: 137 m deep (Dibattista et al. 2016).

Decapods are often studied in less ecological detail than fish (Cartes and Sarda 1993), despite being an abundant and important element of the Mediterranean deep-sea communities (Cartes 1998). *Aristeus antennatus* (the blue and red shrimp) is a large and abundant deep-sea shrimp, making it important to fisheries (Cartes and Sardà 1989). *Aristeus antennatus* was responsible for a large proportion of within group similarity < 1000 m depth (18.79–24.16%; Tables 3 and 4), but was not observed deeper, explaining its absence from the Jones et al. (2003) study, which sampled > 1503 m depth and the ROV survey of Gates et al. (2012) at 2720 m depth. *Aristeus antennatus* was reported in Linley (2012, 2017b) at 642–670 m. *Aristeus antennatus* is, however, known from 2266 m in the western Mediterranean (Cartes 1993) and is understood to vary in distribution seasonally (Cartes and Sardà 1989). Cartes (1994) found a dietary shift in *A. antennatus* at 1200 m depth, which may also represent a shift in response to bait. Cartes and Sardà (1989) found the stomach contents of *A. antennatus* to be predominantly invertebrate prey with bivalves becoming increasingly important and Ophiuroids becoming less important components as the animal grew larger. Fish were found to be a small component of their diet and may suggest a scavenging component. However, the prey species identified are all benthic, and pelagic organisms would also be expected if *A. antennatus* were necrophagous (Cartes and Sardà 1989; Cartes 1998).

Only one species of fish, the macrourid *Coryphaenoides mediterraneus,* and one species of crustacean, the shrimp *Acanthephyra eximia*, were observed at abyssal depths (> 3000 m) in the current study, with a new depth record of 5111 m for the former. In the Eastern Hellenic arc, Jones et al. (2003) also found *C. mediterraneus* to be the only demersal fish species at abyssal depths. C*oryphaenoides mediterraneus* was also the dominant demersal abyssal species both in number and weight in the deep trawl survey of the Ionian Sea (D’Onghia et al. 2004). Observation of both fish and shrimps at 5111 m confirms the presence of mobile megafauna at maximum depth even in the unusually warm and oligotrophic conditions of the Mediterranean Sea. The trend in *C. mediterraneus* of longer first arrival time and lower *N*_max_ with increasing depth indicates a very low population density at maximum depth (Priede and Merrett 1998) equivalent to about 25 fish km^−2^ or 3000–4000 individuals in the whole of the area of the Calypso Deep beyond 5000 m. *Coryphaenoides mediterraneus* is much smaller in the Mediterranean (ca. 15 cm total length in this study) than in the North Atlantic Ocean, where it can reach 73 cm (Geistdoerfer 1986). A reduction in the maximum size in the Mediterranean relative to Atlantic populations has been observed in multiple species (Carrassón et al. 1992; Cartes and Sarda 1993; Cartes et al. 2015). Catarino et al. (2015) show that the shark *Centroscymnus coelolepis* in the Mediterranean has become genetically isolated from populations in the Atlantic Ocean and may have become specialised to the prevailing conditions. *Coryphaenoides mediterraneus* in the Mediterranean Sea may be genetically isolated from their conspecifics in the NE Atlantic by a bathymetric barrier to gene flow at the Straits of Gibraltar and have developed a smaller phenotype. In general, fish size beyond 1000 m depth decreases with increasing depth (Stefanescu et al. 1992). Growth is presumably stunted in the Eastern Mediterranean owing to food limitation (D’Onghia et al. 2004; Wei et al. 2010). Collins et al. (2005) found that in the Atlantic, necrophagous species tended to increase in size with increasing depth, while the inverse was true in the remaining fish species. Overall, there is an increase in fish size with depth in the Atlantic, both generally and at the species level (Mindel et al. 2016); however, the opposite is reported in the western Mediterranean Sea (Stefanescu et al. 1992), where necrophagy does not appear to be an important feeding mode in the fish observed > 1000 m depth. The lower rate at which *t*_arr_ increased with increasing depth in the Mediterranean relative to the Atlantic may be the result of the reduction in fish size compensating for the reduction in biomass, as *t*_arr_ is a proxy for animal density rather than biomass.

The rate of increase in *t*_arr_ and, therefore, decrease in estimated bait-attending fish density with increasing depth, differed for the Mediterranean and Atlantic data sets (Table 5). However, both were of the same order of magnitude found by the global assessment of megafauna biomass (− 0.000307 Log_10_ mg C m^−2^ per m depth) and abundance (− 0.000228 Log_10_ individuals m^−2^ per m depth) by Wei et al. (2010). These depth trends have been further demonstrated in global benthopelagic plankton biomass (− 0.00034 Log_10_ g 1000 m^−3^ per m depth) as well as bioluminescent zooplankton, where near seafloor densities in the Atlantic Ocean (− 0.000546 Log_10_ bioluminescent sources m^−3^ per m depth) were 8 times higher than those of the Mediterranean Sea (− 0.000622 Log_10_ bioluminescent sources m^−3^ per m depth), although rates of decrease with depth were not significantly different (Craig et al. 2015). Measurements of number of bioluminescent targets as a proxy for pelagic abundance provide evidence for the low density of organisms throughout the water column in the eastern compared to the western Mediterranean Sea, where values are typically 2–10 times higher, and in the north Atlantic Ocean, where pelagic densities are an order of magnitude greater (500–5000 m depth; Priede et al. 2008; Craig et al. 2011, 2015).

Deep-sea communities are thought to be primarily food limited (Gage and Tyler 1991; Ruhl et al. 2008; Smith et al. 2008). Surface primary productivity in the oligotrophic eastern basins of the Mediterranean Sea is lower and experiences less seasonal and inter-annual variation than the western Mediterranean (Bricaud et al. 2002; Moutin and Raimbault 2002; Bosc et al. 2004; D’Ortenzio and Ribera d’Alcalà 2008). Productivity in surface layers is exported into deeper water supporting deep-sea populations (Tselepides and Eleftheriou 1992; Riaux-Gobin et al. 2004; Guidi-Guilvard et al. 2007; Papiol et al. 2013). In the Eastern Mediterranean, the fraction of primary production exported below 2000 m depth is 0.3% of the already meagre 145 g C m^−2^ year^−1^ net particulate primary production (Gogou et al. 2014). Low levels of exported organic material from the surface and its rapid bacterial degradation due to the high water temperatures throughout the water column (Laws et al. 2000; Tselepides et al. 2000) result in the relatively low densities of megafauna (Cartes et al. 2004; Massutí et al. 2004), macrofauna (Kröncke et al. 2003), and meiofauna (Danovaro et al. 2000, 2010) in the Mediterranean compared to the Atlantic Ocean. Furthermore, the dominance of highly mobile predatory decapods within the deep Mediterranean invertebrate assemblages indicates a differing optimal feeding strategy in an environment with less particulate organic carbon input, in comparison with the Atlantic, where specialised suspension feeders and echinoderms dominate the deep sea (Cartes and Sardà 1992; Cartes et al. 2004). In the northwest Mediterranean, Fanelli et al. (2011) found that fresh food was only available to suspension feeders for a short period after a phytoplankton bloom and that they relied on resuspended material for the rest of the time. All of the deployments within the current work occurred outside of the spring phytoplankton bloom (Fanelli et al. 2011) which may explain the very clean appearance of the sediment at depth.

Since 2005 commercial trawling has been banned in the Mediterranean Sea at depths greater than 1000 m (Garcia et al. 2014), which means that data for the deep basins will only be obtained through specific scientific studies. Long-term monitoring via cabled observatories may complement scientific trawling by providing temporally high-resolution data of a specific faunal boundary (which are likely to shift temporally). Through the combined analysis presented herein, and its agreement with the wider literature, the placement of long-term observatories at specific faunal depth boundaries is possible. An observatory will not be limited to bait-attending fauna, or differences in bait response, and may offer a more complete picture of the community. The Mediterranean Sea is an environmentally distinct marine habitat, increasingly so toward the east. The cost-effective placement of networked observatories on existing infrastructure provides an opportunity to explore the adaptations of marine fauna to extreme conditions, particularly those relating to energy budgets and optimal feeding strategies. It also provides continued monitoring of the sea’s colonisation via the Suez Canal and likely future environmental changes (Lejeusne et al. 2010).

## Electronic supplementary material

Below is the link to the electronic supplementary material.

Supplementary material 1 (PDF 554 kb)
