# Supplementary material for: Bathyal and abyssal demersal bait-attending fauna of the Eastern Mediterranean Sea
Source: Mar Biol. 2018 Sep 21;165(10):159. doi: 10.1007/s00227-018-3413-0 (PMC6153865; doi:10.1007/s00227-018-3413-0)
Supplement: Supplementary file 1 — Supplementary material 1 (PDF 554 kb) [file 227_2018_3413_MOESM1_ESM.pdf]

Bathyal and abyssal demersal bait attending fauna of the Eastern Mediterranean Sea

Thomas D. Linley. Jessica Craig, Alan J. Jamieson, Imants G. Priede

Supplementary 1: Similarity percentage analysis (SIMPER) of the species which contributed to the similarity within the faunal groups of the combined Eastern Mediterranean dataset

Species	Average N_{max}	Average similarity	Similarity/SD	Percentage contribution	Cumulative percentage
Depth <943 m. Average similarity: 32.32%					
Aristeus antennatus	0.95	7.81	1.11	24.16	24.16
Conger conger	0.82	7.60	1.14	23.52	47.68
Plesionika heterocarpus	0.84	4.59	0.59	14.20	61.89
Helicolenus dactylopterus	0.60	3.69	0.61	11.42	73.31
Centrophorus granulosus	0.57	3.36	0.38	10.39	83.69
Aristaeomorpha foliacea	0.44	2.07	0.44	6.42	90.11
Mora moro	0.33	1.11	0.30	3.44	93.55
Solenocera membranacea	0.31	0.45	0.17	1.39	94.94
Paromola cuvieri	0.22	0.35	0.17	1.10	96.04
Hexanchus griseus	0.22	0.34	0.17	1.06	97.10
Bathynectes longispina	0.27	0.32	0.17	0.98	98.08
Dalatias licha	0.22	0.31	0.17	0.97	99.06
Depth 1208-1346 m. Average similarity: 66.50%					
Acanthephyra eximia	2.53	29.93	SD=0	45.01	45.01
Nettastoma melanurum	1.73	23.18	SD=0	34.86	79.87
Hexanchus griseus	1.00	13.38	SD=0	20.13	100.00
Depth 1503-2307 m. Average similarity: 66.56%					
Acanthephyra eximia	8.67	39.80	3.81	59.80	59.80
Etmopterus spinax	2.24	12.19	6.71	18.32	78.12
Hexanchus griseus	0.98	4.60	1.78	6.92	85.04
Lepidion lepidion	1.01	4.42	1.08	6.64	91.68
Galeus melastomus	0.76	2.51	0.81	3.78	95.45
Geryon longipes	1.18	2.04	0.43	3.06	98.52
Chaceon mediterraneus	0.64	0.72	0.29	1.09	99.61
Depth > 3396 m. Average similarity: 67.24%					
Acanthephyra eximia	4.32	39.63	5.12	58.93	58.93
Coryphaenoides mediterraneus	2.98	25.17	2.84	37.43	96.37
Chaceon mediterraneus	1.13	2.44	0.32	3.63	100.00

Bathyal and abyssal demersal bait attending fauna of the Eastern Mediterranean Sea

Thomas D. Linley. Jessica Craig, Alan J. Jamieson, Imants G. Priede

Supplementary 2: The full output of the LINKTREE analysis of Eastern Mediterranean baited lander Studies. Letters denote each split in the dendrogram (Fig. 6) and deployments are labelled by depth. Significant splits are in bold.

Split	Left side	Right side	B%	R	Statistic
**A->B,G**	**Depth<943**	**Depth >1210**	**98.6**	**0.96**	**Pi = 6.00, p = 0.001**
B->C, 943 m	Depth<820	Depth>943	88.7	0.83	Pi = 3.79, p = 0.071
C->D,E	Duration<7	Dur>8.4	53.2	0.52	
D->(532m,737 m),(642 m, 651 m)	Lat<36.9, Long>21.6, Dur<6	Lat>39.6, Long<18.5, Dur>6.5	46.6	1.00	
E->820 m,F	Lat<32.5, Long>19.8, Depth>820	Lat>39.6, Long<18.5, Depth<673	51.1	1.00	
F->(661 m, 673 m),(653 m)	Dur<8.6, Depth>667, Lat<39.6, Long<18.5	Dur>34.9, Depth<653, Lat>39.6, Long>18.5	16.2	1.00	
**G->H,N**	**Depth <2310 m**	**Depth >2490 m**	**45.7**	**0.78**	**Pi = 3.56, p = 0.001**
**H->(1208 m, 1346 m),I**	**Depth<1350**	**Depth>1500**	**39.2**	**0.88**	**Pi = 2.71, p = 0.034**
I->J,(1841 m)	Lat<36, Dur>10.7	Lat>36.9, Dur<3.2	27.5	0.84	Pi = 1.52, p = 0.398
J->(1503 m),K	Depth<1500	Depth>1750	15.6	0.50	
K->L,(2220,2307)	Long<25.2, Depth<2210	Long>26.2, Depth>2220	8.9	0.47	
L->M,(1750 m)	Lat<35.9	Lat>36	8.9	0.58	
M->(1750 m, 2209 m),(1822 m,1873 m)	Long<18.4, Lat<32.9, Dur<16.2	Long>25.1, Lat>35.8, Dur>48	5.7	0.75	
N->O,(5111 m)	Depth<4260	Depth>5110	20.1	0.59	Pi = 1.89, p = 0.485
O->(2490 m, 3080 m),P	Depth<3080	Depth>3400	16.1	0.75	
P->(3396 m),Q	Long<15.8, Dur<1, Depth<3400	Long>21.5, Dur>17.3, Depth>3850	10.6	0.83	
Q->R,(3850 m)	Long<26.2, Depth>4170	Long>28.4, Depth<3850	5.7	0.78	
R->(4172 m, 4264 m),(4204 m)	Dur>67, Long>26.1, Lat<34.4	Dur<17.3, Long<21.5, Lat>36.6	2.0	1.00	
